# Supplementary material for: Cryo-EM analyses unveil details of mechanism and targocil-II mediated inhibition of S. aureus WTA transporter TarGH
Source: Nat Commun. 2025 Apr 4;16:3224. doi: 10.1038/s41467-025-58202-w (PMC11971408; doi:10.1038/s41467-025-58202-w)
Supplement: Supplementary file 1 — Supplementary Information [file 41467_2025_58202_MOESM1_ESM.pdf]

Supplementary Materials for

Cryo-EM analyses unveil details of mechanism and targocil-II mediated inhibition of *S. aureus* WTA transporter TarGH

Li, F.K.K. *et al.*

*Corresponding author. Email: ncjs@mail.ubc.ca

This PDF file includes:

Supplementary Tables 1 to 2

Supplementary Figs. 1 to 9

Protein conformation:	TarGH D-loop$_{OFF}$	TarGH D-loop$_{OFF}$	TarGH D-loop$_{OFF}$	TarGH D-loop$_{ON}$	TarGH D-loop$_{ON}$
**Ligands:**	ATPγS	ATPγS	ATPγS + targocil-II	ATPγS + targocil-II	AMP-PNP + targocil-II
**Expression Source:**	*E. coli* produced	*L. lactis* produced	*L. lactis* produced	*L. lactis* produced	*L. lactis* produced
**Accession codes:**	EMBD-45550<br>PDB: 9CFL	EMBD-48274<br>PDB: 9MHD	EMBD-48282<br>PDB: 9MHZ	EMBD-48281<br>PDB: 9MHU	EMBD-45554<br>PDB: 9CFP
**Data collection and processing**					
Collection location	PNCC	HRMEM	HRMEM	HRMEM	PNCC
Microscope	TF Krios G3	TF Krios G2	TF Krios G2	TF Krios G2	TF Krios G3
Camera	Gatan K3	Falcon 4i	Falcon 4	Falcon 4i	Gatan K3
Voltage (kV)	300	300	300	300	300
Defocus range (μm)	0.5-2.0	0.5-2.0	0.5-2.0	0.5-2.0	0.5-2.0
Movies	6,639	8,888	23,286	23,286	37,847
Physical pixel size (Å)	0.53	0.59	0.59	0.59	0.53
Total dose (e–/Å^2)	50	50	50	50	50
Symmetry imposed	C2	C2	C2	C2	C2
Particles initial / final	2,906,596 / 153,452	696,797 / 61,002	2,331,724 / 121,566	2,331,724 / 113,338	6,924,662 / 117,415
Map resolution (Å)	2.3	2.9	2.7	3.0	2.9
FSC threshold	0.143	0.143	0.143	0.143	0.143
**Model Refinements**					
Initial model used (PDB code)	6JBH	9CFL	9CFL	9CFL	9CFL
Model resolution (Å)	2.5	3.3	2.9	3.3	3.2
FSC threshold	0.5	0.5	0.5	0.5	0.5
Map sharpening B-factor (Å^2)	-88.1	-97.3	-88.8	-108.9	-103.3
**Model composition**					
Non-hydrogen atoms	8925	8923	8924	8786	8806
Protein	1068	1038	1068	1068	1068
Ligands	7	7	8	6	6
Mean Model B-factors (Å2)					
Protein	43.08	62.74	46.41	52.61	61.50
Ligand	55.68	76.09	54.07	45.32	59.06
R.m.s. deviations					
Bond lengths (Å)	0.008	0.003	0.005	0.005	0.003
Bond angles (°)	1.421	0.499	0.718	0.653	0.520
Validation					
MolProbity score	1.61	1.76	1.74	1.96	1.80
All-atom clash score	3.76	4.37	3.28	6.85	4.25
Rotamer outliers (%)	3.83	4.36	4.15	3.16	4.14
C-beta deviations (%)	0.20	0.00	0.00	0.00	0.00
Ramachandran plot					
Favoured (%)	98.87	97.74	97.17	96.79	97.37
Allowed (%)	1.13	2.26	2.83	3.21	2.63
Disallowed (%)	0.00	0.00	0.00	0.00	0.00

Supplementary Table 1. *S. aureus* TarGH data collection and processing statistics.

Probe	Forward Primer (5' – 3')	Reverse Primer (5' – 3')
***Construct 1*- pETDuet insertion**		
MCS1- TarG (codon optimized)	GTTTAACTTTAATAAGGAGATATACCATGGGCA GCAGCCATCATCACCATCATCACC	CGATTACTTTCTGTTCGACTTAAGCATTACA AAAAGTCGGCGAACTGGTCACGGTATTTC
MCS2- TarH (codon optimized)	GTATAAGAAGGAGATATACATATGAACGTGTCA GTAAATATTAAAAATGTTACAAAG	CTTTCGCGTGGCACCAGAGCCTCGAGTCAT TTAATCACGAAGCGGCTTTCG
***Construct 2*- Generation of pNZDual template (pNZ8048)**		
pNZ8148 MCS1/2 amplification	GACTGGCTTTTATAATATGAGATAATGCCGACT GTACTTTTTACAGTCGGTTTTCT	CAACACGTGCTGTAATTTGTTTAATTGCCAT TTCAATTGAACGTTTCAAGCCTAGG
***Construct 3*- Generation of N-term tagged TarG in pNZ8048**		
TarG (USA300)	CAGCAGCGGCCTGGTGCCGCGCGGCAGCCAT ATGTCAGCAATAGGAACAGTTTTTAAAG	CTTTGGTATTTGATTACTAATACGTTTTACAA GAAGTCTGCAAATTGATCTCTATATTTC
***Construct 4*- Generation of TarGH pNZDual plasmid**		
N-term tagged TarG (USA300)	CAAAATAAATTATAAGGAGGCACTCACCATGGG CAGCAGCCATCATCACCATCATCAC	CGGGGCAGGTTAGTGACATTTCTAGATTACA AGAAGTCTGCAAATTGATCTCTATATTTC
TarH (USA300)	AATAAATTATAAGGAGGCACTCCATATGAACGT TTCGGTAAACATTAAAAATGTAAC	CTAATTTTGGTTCAAAGAAAGCTTTTATTTAA TAACGAAGCGGGACTCATC

Supplementary Table 2. Primers utilised in construct design for *E. coli* and *L. lactis* TarGH expression

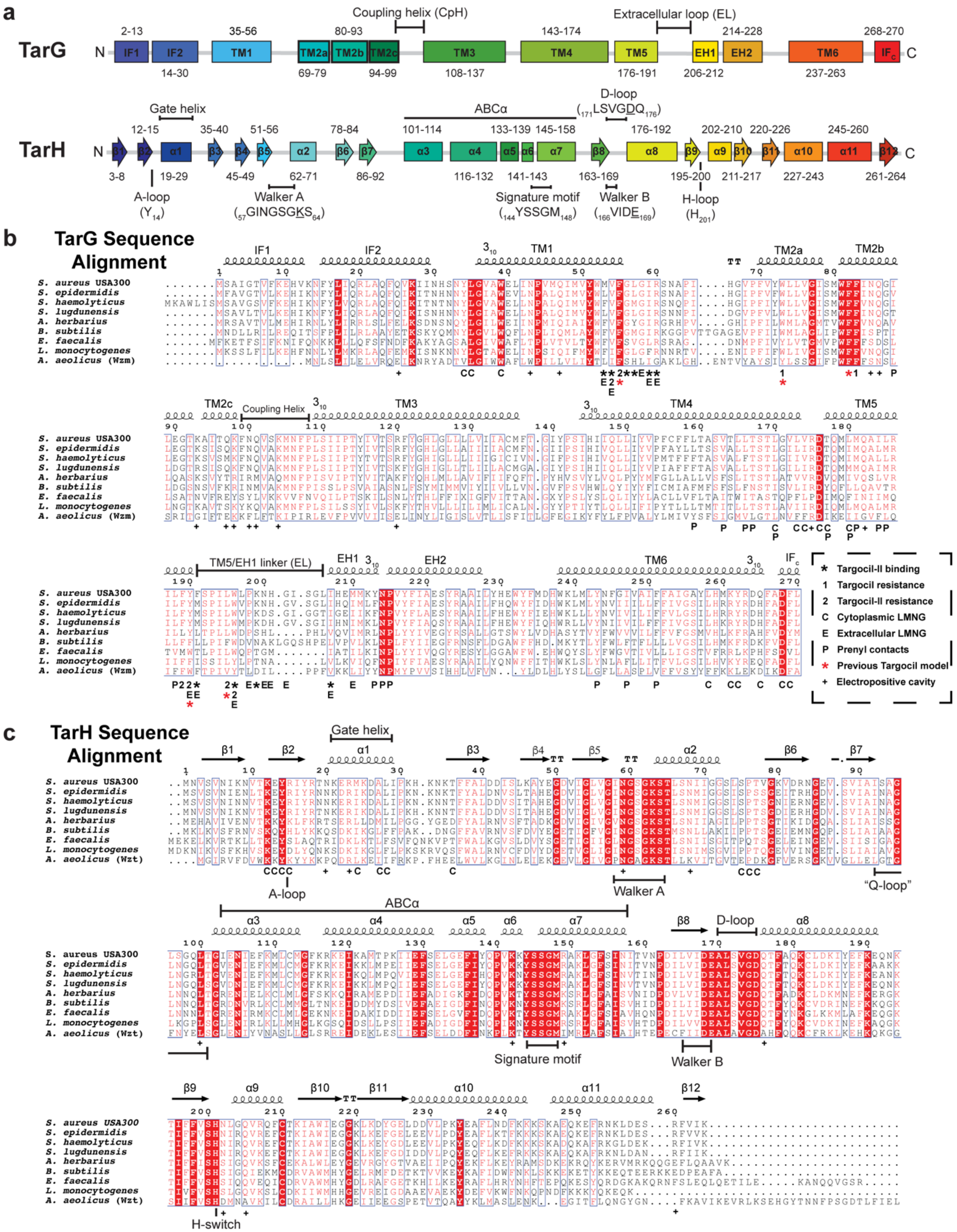

Supplementary Fig. 1: *S. aureus* TarGH secondary structural elements and amino acid sequence alignments. **a** Secondary structure **and sequence motifs** of *S. aureus* TarG and TarH. **b** Amino acid sequence alignment of transmembrane domain TarG orthologues from USA300 *S. aureus* (used in this study; Refseq ID: ABD22558.1), *Staphylococcus epidermidis RP62A* (Refseq ID: WP_001832041.1), *Staphylococcus haemolyticus* (Refseq ID: BAE05567.1), *Staphylococcus lugdunensis* (Refseq ID: TBW71547.1),

Alicyclobacillus herbarius (Refseq ID: WP_026962790.1), *Bacillus subtilis 168* (Refseq ID: WP_003227928.1), *Enterococcus faecalis strain V583* (Refseq ID: AAC35925.1), *Listeria monocytogenes* (Refseq ID: HAC3253105.1). Homologous type V ABC transporter *Aquifex aeolicus* Wzm (Refseq ID: WP_010880683) also included. Secondary structure for *S. aureus* TarG determined here shown above and key residues are indicated below as per key in box. **c** As per **b** for nucleotide binding domain TarH. USA300 *S. aureus* ((Refseq ID: Q2FJ01.1), *Staphylococcus epidermidis RP62A* (Refseq ID: WP_001832071.1), *Staphylococcus haemolyticus* (Refseq ID: WP_011276519.1), *Staphylococcus lugdunensis* ((Refseq ID: WP_002461317.1), *Alicyclobacillus herbarius* TarH (Uniprot: A0A6I8WFL6), *Bacillus subtilis 168* (Refseq ID: WP_003227930.1), *Enterococcus faecalis strain V583* (Refseq ID: WP_010706558.1), *Listeria monocytogenes* (Refseq ID: HAA8326601.1), *and Aquifex aeolicus* Wzt (Refseq ID: *WP_010880682.1*).

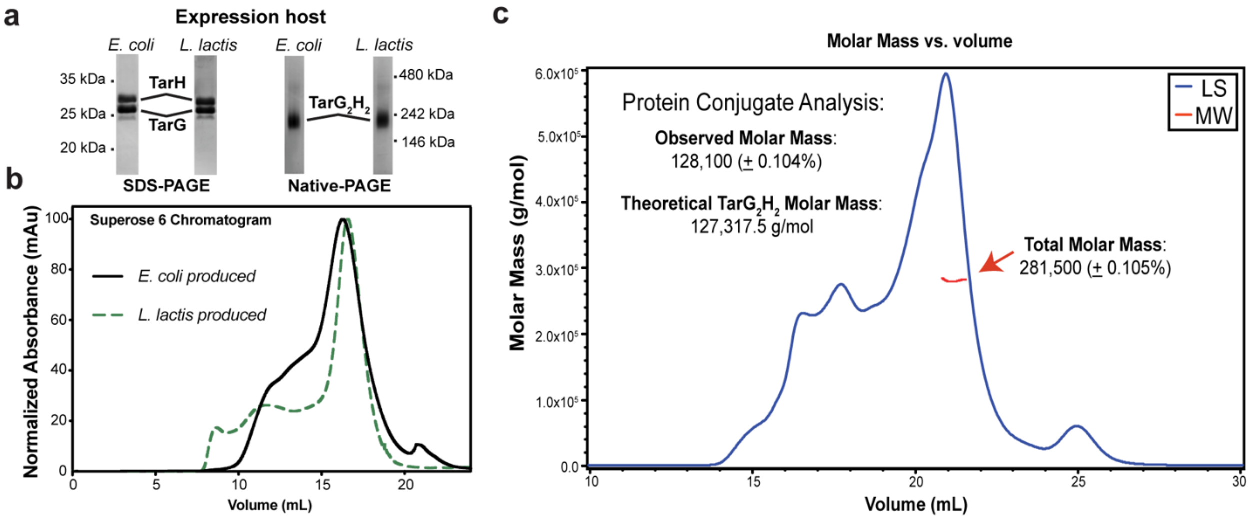

Supplementary Fig. 2: *In vitro* characterization by poly-acrylamide gel electrophoresis (PAGE), size-exclusion chromatography, and size-exclusion chromatography tandem multi-angle light scattering (SEC-MALS) of LMNG-extracted *S. aureus* TarGH. a Purity of purified *S. aureus* TarGH utilized for functional assays and cryo-EM. Sample run under denaturing (SDS-PAGE) and native (Native-PAGE) conditions. **b** Size exclusion chromatography using a Superose 6 Increase, 10/300 GL column shows *S. aureus* TarGH elutes as a single peak from either *E. coli* or *L. lactis* produced protein. **c** SEC-MALS analysis of *L. lactis* produced *S. aureus* TarGH. Protein conjugate analysis of the LMNG extracted TarGH shows a heterotetramer composed of two TarH and two TarG protomers. The chromatogram (blue line) represents light scattering with the red line indicating the molar mass (left axis). Total molar mass corresponds to the entire TarGH/LMNG complex and observed molar mass represents the detergent corrected molar mass after protein conjugate analysis was applied. A LMNG dn/dc value of 0.14 ml/g and ProtParam generated TarGH heterotetrametric extinction coefficient were used.

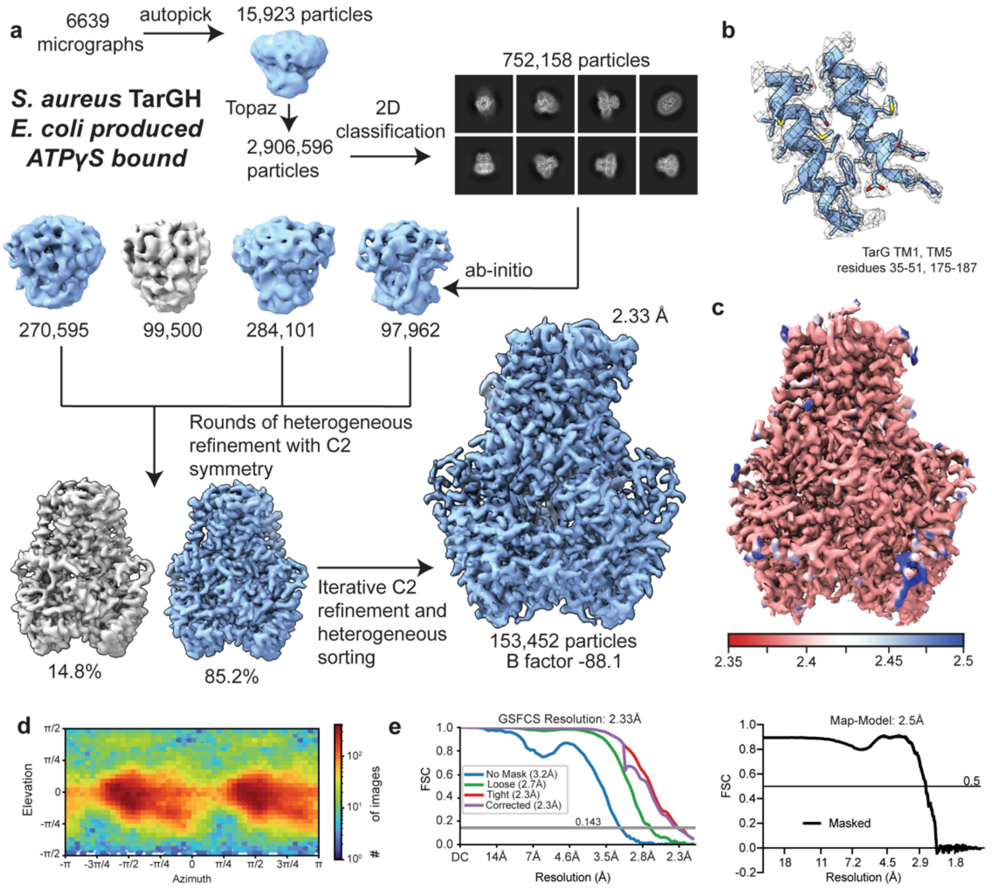

Supplementary Fig. 3: Single particle cryo-EM data processing of *E. coli-expressed,* LMNG extracted *S. aureus* TarGH in complex with Mg^{2+} and ATPγS. **a** Processing workflow was completed in cryoSPARC[1] with representative cryo-EM density maps modeled in ChimeraX[2]. **b** Example density for *S. aureus* TarG ATPγS-bound secondary structural elements shown in dark grey mesh (map-threshold of 0.15). **c** Cryo-EM density map as in **a** coloured by local resolution. **d** Particle orientation distribution heat map. **e** Gold-standard half-map FSC curves with 0.143 cutoff threshold. **f** Gold-standard model-map FSC curve with 0.5 FSC cutoff threshold.

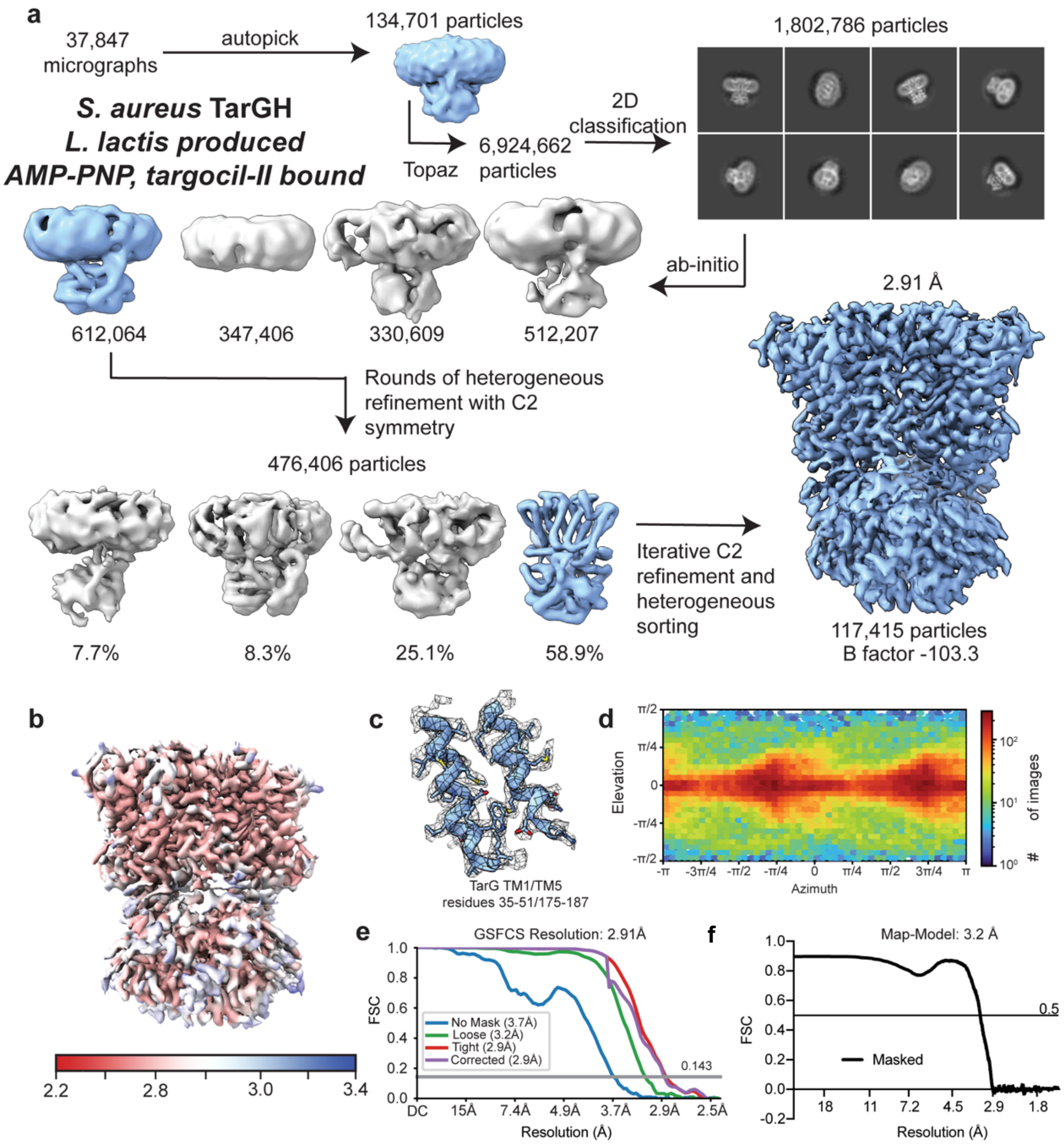

Supplementary Fig. 4: Single particle cryo-EM data processing of *L. lactis-expressed,* LMNG extracted *S. aureus* TarGH in complex with Mg^{2+}, AMP-PNP, and targocil-II. **a** Processing workflow was completed in cryoSPARC[1] with representative cryo-EM density maps modeled in ChimeraX[2]. **b** Refined cryo-EM density map as in **A** colored by local resolution. **c** Example density for targocil-II inhibited *S. aureus* TarG AMP-PNP bound secondary structural elements shown in dark grey mesh (map-threshold of 0.15). **d** Particle orientation distribution heat map. **e** Gold-standard half-map FSC curves with 0.143 cutoff threshold. **f** Gold-standard model-map FSC curve with 0.5 FSC cutoff threshold.

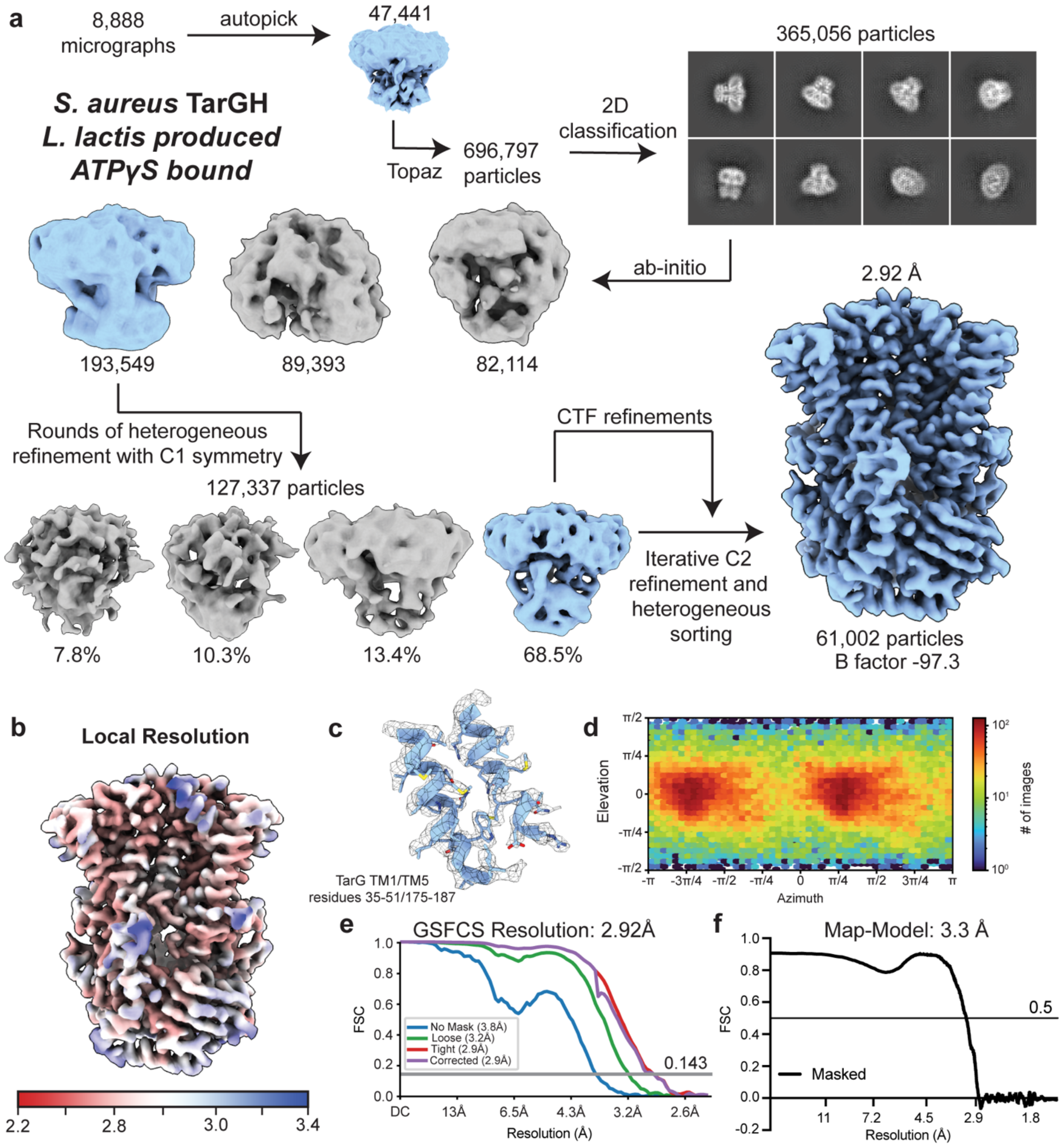

Supplementary Fig. 5: Single particle cryo-EM data processing of *L. lactis-expressed,* LMNG extracted *S. aureus* TarGH in complex with Mg^{2+} and ATPγS. a Processing workflow was completed in cryoSPARC[1] with representative cryo-EM density maps modeled in ChimeraX[2]. **b** Refined cryo-EM density map as in **a** colored by local resolution. **c** Example density for *S. aureus* TarG ATPγS-bound secondary structural elements shown in dark grey mesh (map-threshold of 0.15). **d** Particle orientation distribution heat map. **e** Gold-standard half-map FSC curves with 0.143 cutoff threshold. **f** Gold-standard model-map FSC curve with 0.5 FSC cutoff threshold.

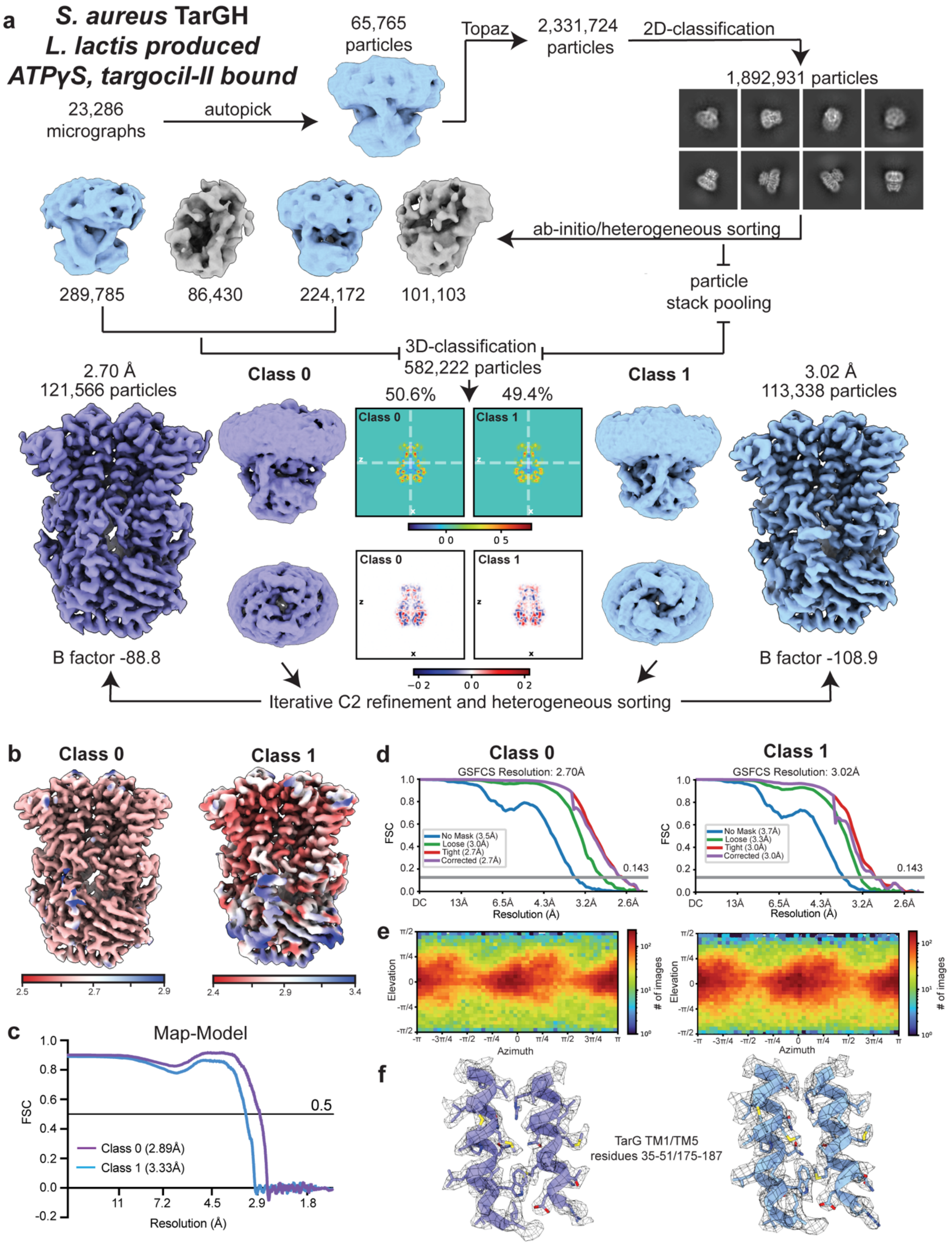

Supplementary Fig. 6: Single particle cryo-EM data processing of *L. lactis-expressed,* LMNG extracted *S. aureus* TarGH in complex with Mg^{2+}, ATPγS, and targocil-II. a Processing workflow was completed in cryoSPARC[1] with representative cryo-EM

density maps modeled in ChimeraX[2]. Class 0 represents the D-loop$_{OFF}$ conformation while Class 1 represents the D-loop$_{ON}$ conformation. **b** Refined cryo-EM density map as in **a** colored by local resolution form each isolated class. **c** Gold-standard half-map FSC curves with 0.143 cutoff threshold. **d** Gold-standard model-map FSC curves with 0.5 FSC cutoff threshold. **e** Particle orientation distribution heat map. **f** Example density for targocil-II inhibited *S. aureus* TarG AMP-PNP bound secondary structural elements shown in dark grey mesh (map-threshold of 0.15).

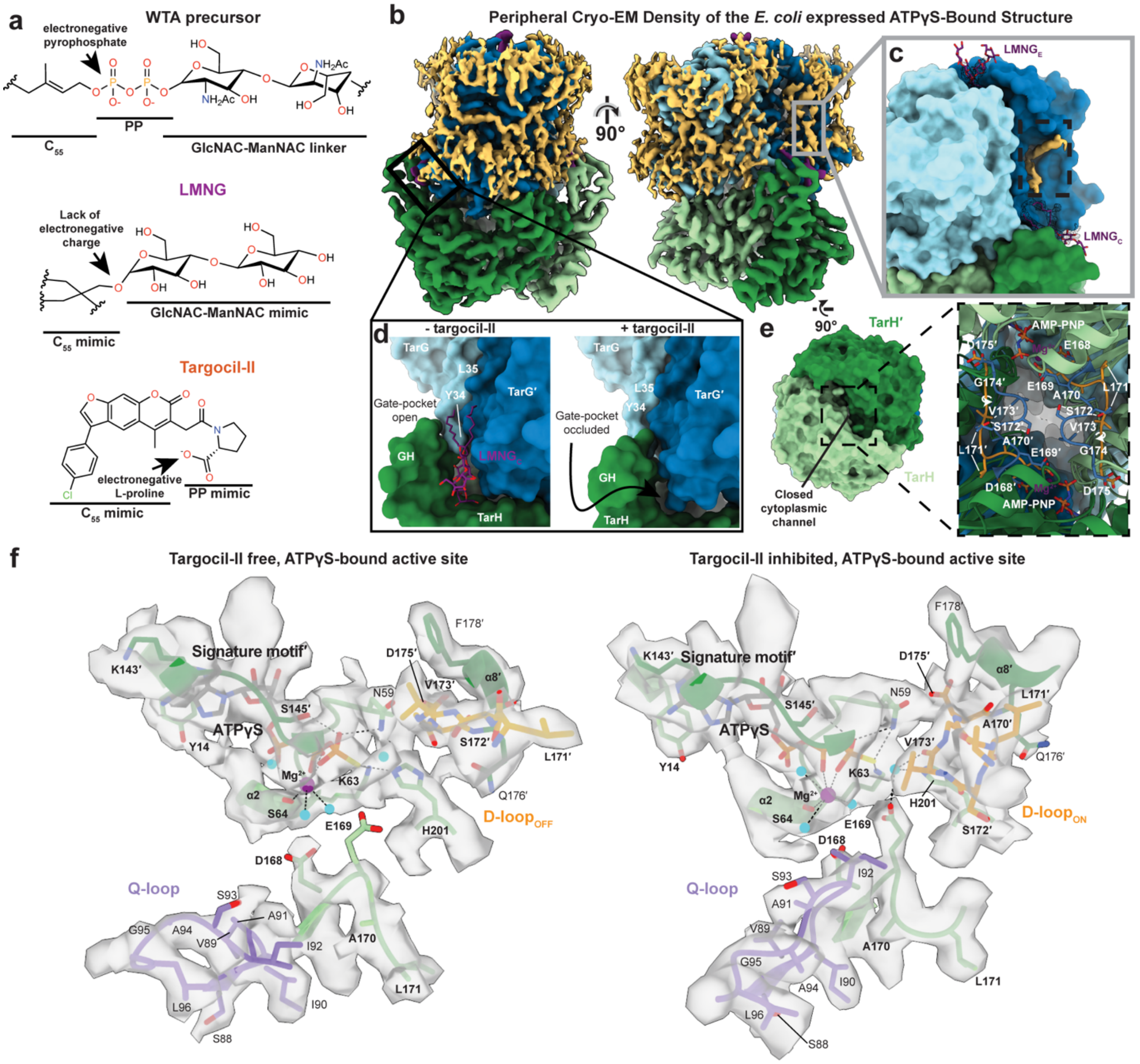

Supplementary Fig. 7: Structural features and observed differences across reconstructed *S. aureus* TarGH models. a Chemical structure and comparison of structurally resolved ligands (LMNG and targocil-II) to native TarGH substrate. Ligand structural motifs are characterized by putative structural similarity to WTA-precursor (middle). **b** Non-protein density in the 2.3 Å resolution TarGH structure. TarG and TarH are coloured blue/green, respectively with additional map densities shown in gold (map-threshold of 0.2). Boxed region shown in **c**. **c** TarGH surface depiction with the most defined ordered lipid density (map-threshold 0.19, surface depiction) located below the TarG EH1/2 reentrant helices. LMNG, shown as purple sticks with heteroatom colouring, are surrounded by a mesh map-density at the same threshold for reference. **d** Close up view of the substrate binding site defined by the TarH GH shown on in absence (left) and presence (right) of bound targocil-II. LMNG$_C$ binds the pocket in absence of targocil-II (purple sticks), we propose mimicking substrate. Targocil-II binding leads to a translation of the GH and collapse of the binding pocket with no LMNG observed. **e** Surface view of the from base of TarGH illustrating the closed channel in the D-loop$_{ON}$ conformation. Boxed region provides a zoomed-in perspective of the D-loop movement from the D-loop$_{OFF}$ (orange) to D-loop$_{ON}$ (blue) conformations. TarG is depicted as a surface with TarH shown as cartoon ribbons, coloured as in **b**. **f** Cryo-EM density around TarH active site in absence (left; D-loop$_{OFF}$ at 2.3 Å resolution) and presence (right; D-loop$_{ON}$ at 2.9 Å resolution). Density shown as a transparent grey surface with a map-threshold of 0.35 and 0.22 for maps in the absence and presence of targocil-II, respectively). TarH E169 corresponding density is apparent in the targocil-II density map but not ATPγS alone.

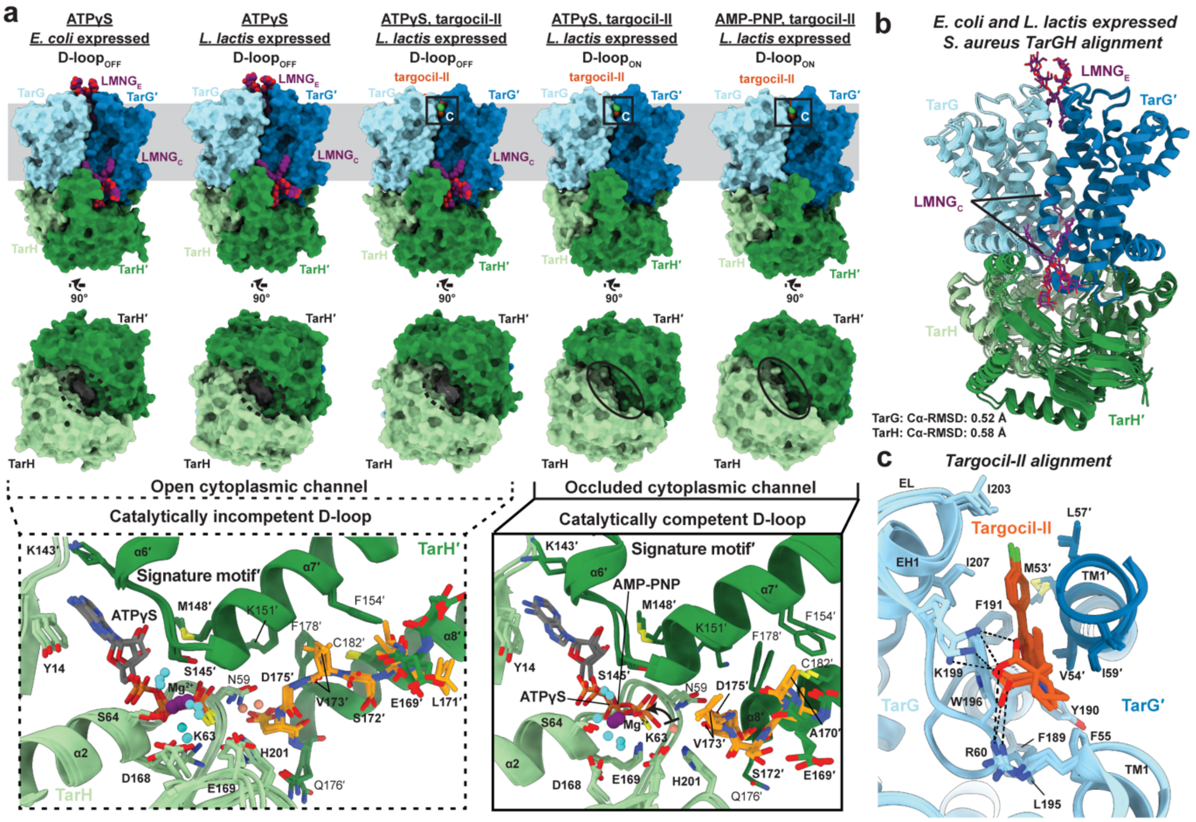

Supplementary Fig. 8: Structural comparison of resolved TarGH structures and ligands. a Surface representation of the five *S. aureus* TarGH structures determined here (top) with bound ligands (LMNG and targocil-II) shown as purple and orange spheres, respectively, with heteroatom colouring. Cytoplasmic perspective and the corresponding D-loop conformations are shown at the bottom, with the boxed-out regions showing superposed D-loop$_{OFF}$ (dashed) or D-loop$_{ON}$ (solid) active sites. TarH ABC motif residues are shown as sticks, with nucleotides (ATPγS and AMP-PNP) in grey, coordinated water molecules in cyan, catalytic water in salmon, and magnesium in purple and depicted as a sphere. The D-loop of the opposing TarH protomer is coloured orange with heteroatom colouring and represented as sticks. **b** Ribbon overlay of the *E. coli expressed* and *L. lactis* expressed *S. aureus* TarGH structures with bound LMNG shown as sticks, coloured purple with heteroatom colouring. The relative Cα RMSDs are listed below. **c** Ribbon overlay of the TarG extracellular targocil-II binding pocket from targocil-II bound TarGH with AMP-PNP (D-loop$_{ON}$), ATPγS-bound (D-loop$_{ON}$), and ATPγS-bound (D-loop$_{OFF}$). Direct interaction of the L-proline carboxylate with R60 and K199 are shown as dashed lines with the binding pocket and interacting residues shown as sticks and coloured light/dark blue with heteroatom colouring.

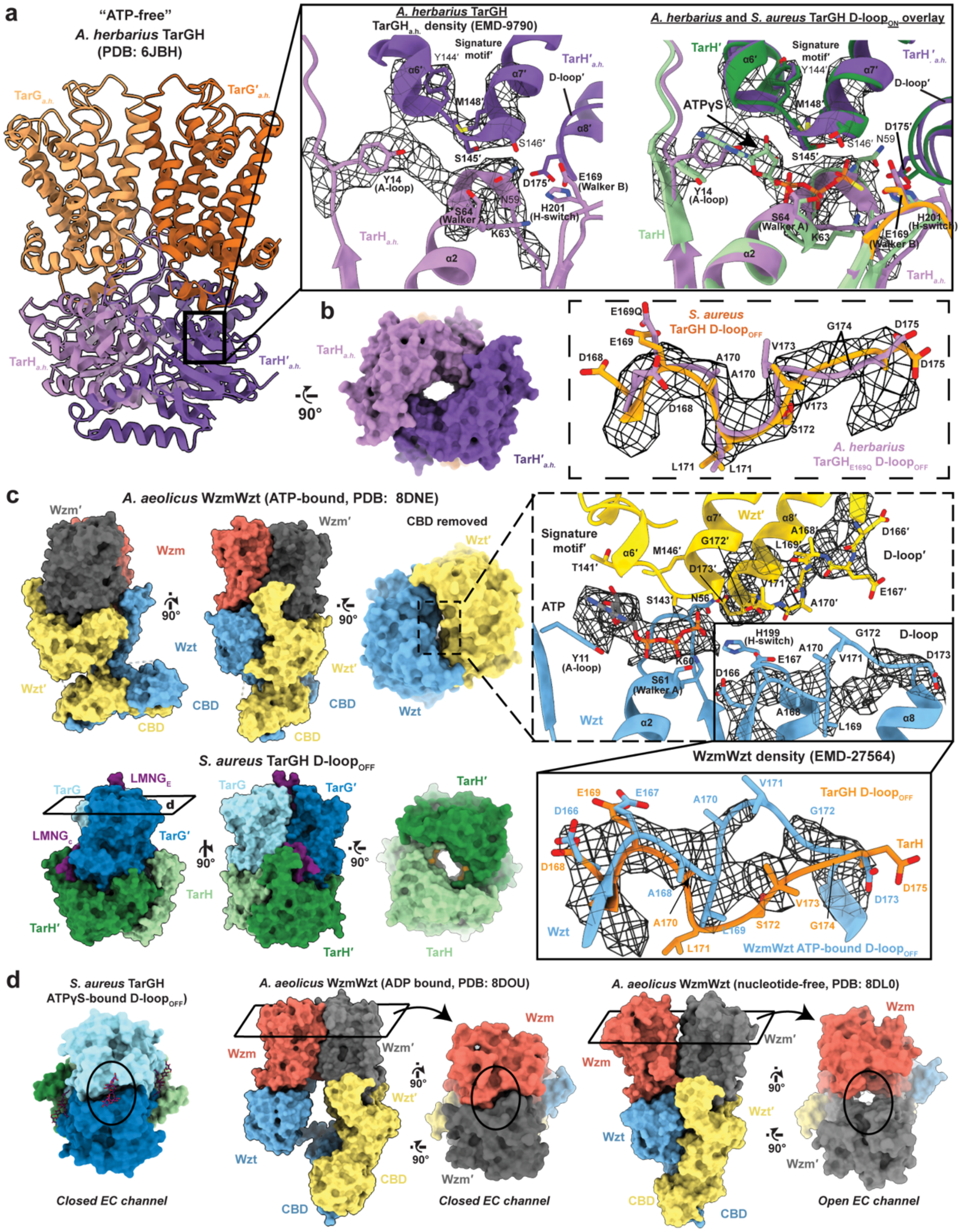

Supplementary Fig. 9: Structural comparisons of bacterial ABC transporter Type V lipid-transferases. a Cryo-EM structure of the *Alicyclobacillus herbarius* TarGH E169Q catalytically mutant[3] (PDB: 6JBH) shown as ribbon with TarG coloured light/dark orange and TarH coloured light/dark purple. Boxed region shows close up of the TarH active site modelled without bound nucleotide as deposited (left) with cryo-EM density (dark grey mesh; EMD-9790, map-threshold of 0.13) shown around the A-loop, signature motif, and Walker A motif. A superposed view with the ATPγS-bound TarGH D-loop$_{OFF}$ (green) determined here is shown on right showing the density supports a bound endogenous nucleotide, likely ATP. **b** Bottom-up view of **a** in surface representation showing similar open channel within the TarH dimer defined by the D-loop$_{OFF}$ conformation. Boxed region shows close up view of the D-loop from the *A. herbarius* structure (purple; corresponding cryo-EM density shown as grey mesh at a map-threshold of 0.1) and ATPγS-bound TarGH D-loop$_{OFF}$ determined here (gold). **c** Comparison of the ATPγS-bound TarGH D-loop$_{OFF}$ structure (bottom) with the *Aquifex aeolicus* WzmWzt ATP-bound structure[4] (top) determined from a sample undergoing ATP hydrolysis (incubated with ATP for one hour prior to freezing). View in solid box shows close up of Wzt NBD active site dimer with cryo-EM density shown in grey mesh (EMD-27564, map-threshold of 0.18). Dashed box further highlights the modelled D-loop conformation (blue) with supporting density at the same threshold. The D-loop$_{OFF}$ from the ATPγS-bound TarGH structure is overlaid in orange suggesting both the D-loop$_{ON}$ (as

modelled) and D-loop$_{OFF}$ conformations are likely present in this active sample. **d** Conformational variability within the transmembrane Wzm domains. The *S. aureus* TarGH ATPγS D-loop$_{OFF}$ structure (left), *Aquifex aeolicus* WzmWzt ADP-bound “teepee” structure (EMD-27623; middle) in an NDB splayed state of the NBDs with TMD interface open up to conserved aromatic constriction, and nucleotide-free, outward open state (EMD-27494; right) are shown as surface models with side (WzmWzt only) and extracellular perspectives. Given the structural homology between WzmWzt and TarGH, similar states likely exist and may inform substrate translocation.

Supplementary References:

1 Ali Punjani, J. L. R. D. J. F. M. A. B. cryoSPARC- algorithms for rapid unsupervised cryo-EM structure determination.pdf. *Nature Methods* **14** (2017). https://doi.org/10.1038/nmeth.4169nature

2 Goddard, T. D. *et al.* UCSF ChimeraX: Meeting modern challenges in visualization and analysis. *Protein Sci* **27**, 14-25 (2018). https://doi.org/10.1002/pro.3235

3 Chen, L. *et al.* Cryo-electron Microscopy Structure and Transport Mechanism of a Wall Teichoic Acid ABC Transporter. *mBio* **11** (2020). https://doi.org/10.1128/mBio.02749-19

4 Spellmon, N. *et al.* Molecular basis for polysaccharide recognition and modulated ATP hydrolysis by the O antigen ABC transporter. *Nat Commun* **13**, 5226 (2022). https://doi.org/10.1038/s41467-022-32597-2
